# Thymol modulates the endocannabinoid system and gut chemosensing of weaning pigs

**DOI:** 10.1186/s12917-020-02516-y

**Published:** 2020-08-12

**Authors:** Andrea Toschi, Benedetta Tugnoli, Barbara Rossi, Andrea Piva, Ester Grilli

**Affiliations:** 1 Department of Veterinary Medical Sciences, University of Bologna, Via Tolara di Sopra, 50, 40064 Ozzano dell’Emilia, BO Italy; 2 Vetagro SpA, via Porro, 2, 42124 Reggio Emilia, Italy; 3 Vetagro, Inc., 116 W. Jackson Blvd, Chicago, IL 60604 USA

**Keywords:** Endocannabinoid system, Gut chemosensing, Inflammation, Pig, Thymol, Small intestine

## Abstract

**Background:**

The recent identification of the endocannabinoid system in the gastrointestinal tract suggests a role in controlling intestinal inflammation. In addition, the gut chemosensing system has therapeutic applications in the treatment of gastrointestinal diseases and inflammation due to the presence of a large variety of receptors. The purposes of this study were to investigate the presence of markers of the endocannabinoid system and the chemosensing system in the pig gut and, second, to determine if thymol modulates these markers. One hundred sixty 28-day-old piglets were allocated into one of 5 treatment groups (*n* = 32 per treatment): T1 (control), T2 (25.5 mg thymol/kg feed), T3 (51 mg thymol/kg feed), T4 (153 mg thymol/kg feed), and T5 (510 mg thymol/kg feed). After 14 days of treatment, piglets were sacrificed (*n* = 8), and then duodenal and ileal mucosal scrapings were collected. Gene expression of cannabinoid receptors (CB1 and CB2), transient receptor potential vanilloid 1 (TRPV1), the olfactory receptor OR1G1, diacylglycerol lipases (DGL-α and DGL-β), fatty acid amine hydrolase (FAAH), and cytokines was measured, and ELISAs of pro-inflammatory cytokines levels were performed.

**Results:**

mRNAs encoding all markers tested were detected. In the duodenum and ileum, the CB1, CB2, TRPV1, and OR1G1 mRNAs were expressed at higher levels in the T4 and T5 groups compared to the control group. The level of the FAAH mRNA was increased in the ileum of the T4 group compared to the control. Regarding the immune response, the level of the tumor necrosis factor (TNF-α) mRNA was significantly increased in the duodenum of the T5 group, but this increase was not consistent with the protein level.

**Conclusions:**

These results indicate the presence of endocannabinoid system and gut chemosensing markers in the piglet gut mucosa. Moreover, thymol modulated the expression of the CB1, CB2, TRPV1, and OR1G1 mRNAs in the duodenum and ileum. It also modulated the mRNA levels of enzymes involved in the biosynthesis and degradation of endocannabinoid molecules. Based on these findings, the effects of thymol on promoting gut health are potentially mediated by the activation of these receptors.

## Background

The endocannabinoid system (ECS) comprises three fundamental constituents: receptors, signaling molecules, and enzymes involved in ligand biosynthesis and degradation. The main endocannabinoid receptors are two G protein-coupled receptors (GPCRs) named cannabinoid receptor 1 (CB1) and cannabinoid receptor 2 (CB2) [1, 2], but additional receptors may also be involved, such as transient receptor potential vanilloid 1 (TRPV1) [3]. The two most widely investigated endocannabinoid signaling molecules are anandamide (AEA) and 2-arachidonoylglycerol (2-AG), which are lipid molecules generated from the breakdown of arachidonic acid [4]. N-Acylphosphatidylethanolamine-specific phospholipase D (NAPE-PLD) is currently considered the major enzyme responsible for AEA synthesis, while a specific diacylglycerol lipase (DGL) is responsible for 2-AG synthesis [4, 5]. The biological activity of ligands is regulated by intracellular enzymes unique to each endocannabinoid ligand: fatty acid amide hydrolase (FAAH) is the principal enzyme responsible for degrading AEA, whereas monoacylglycerol lipase (MAGL) is responsible for degrading 2-AG [6–8]. In non-neuronal tissues, endocannabinoids act as hormone-like messengers in an autocrine or paracrine mode of action, which is thought to be temporally and spatially restricted [9]. When endocannabinoid molecules are released, they bind receptors with different affinity. After the binding of ligands, receptors mediate the effects, including the stimulation of extracellular-regulated kinases and the inhibition of adenylyl cyclase [10]. The ECS controls a variety of gastrointestinal (GI) functions: it is presumed to regulate GI motility via the enteric nervous system (ENS) [11], to inhibit the secretion of pro-inflammatory cytokines and to attenuate the lipopolysaccharide (LPS)-induced increase in GI transit [12]. For example, CB1 receptors are colocalized with acetylcholine transferase, which is a marker for cholinergic neurons. This observation supports the role of endocannabinoids as inhibitors of intestinal motility and secretion by inhibiting cholinergic neurotransmission [10]. For all these reasons, dysregulation of the ECS clearly plays a role in intestinal disorders, including irritable bowel disease (IBD), irritable bowel syndrome, and obesity [13].

Gut chemosensation represents the ability of the gut to sense chemical and nutrient stimuli at the GI level through the action of enteroendocrine cells (EEC) [14], and it appears to be connected to the presence of chemosensory receptors in the mouth and all along the GI tract [15]. This activity is mediated by a large variety of receptors, most of which are GPCRs, including TRP (Transient Receptor Potential) channels and olfactory receptors (ORs). Transient Receptor Potential channels comprise six related protein subfamilies [16] that include TRPV1, which was previously mentioned as one of the secondary endocannabinoid receptors, whereas ORs have a role in recognizing odorant molecules in the olfactory sensory system [17]. Botanicals such as thymol play a role in regulating the integrity of the intestinal mucosa because of their anti-inflammatory and antioxidant properties [18]. In particular, thymol is a monoterpene and it is a predominant component of several essential oils derived from plant species belonging to the *Lamiaceae* family. Thymol showed therapeutic potential in reducing oxidative stress, boosting the immune system and fighting against pathogenic bacteria [19], and it is also well known in animal nutrition as a key component of many botanical feed additives [20]. Therefore, the purposes of this study were 1) to investigate the presence of ECS and gut chemosensory markers in the GI tract of piglets and 2) to evaluate the possible modulation of these systems by treatment with a thymol supplement.

## Results

### Growth performance

Piglets maintained a good health status throughout the experiment and no mortality was recorded. During the experiment, differences in body weight (BW), feed intake (FI), average daily feed intake (ADFI), average daily gain (ADG), and feed to gain ratio (F:G) were not observed among the treatment groups (data not shown).

### Endocannabinoid system

**Fig. 1:**
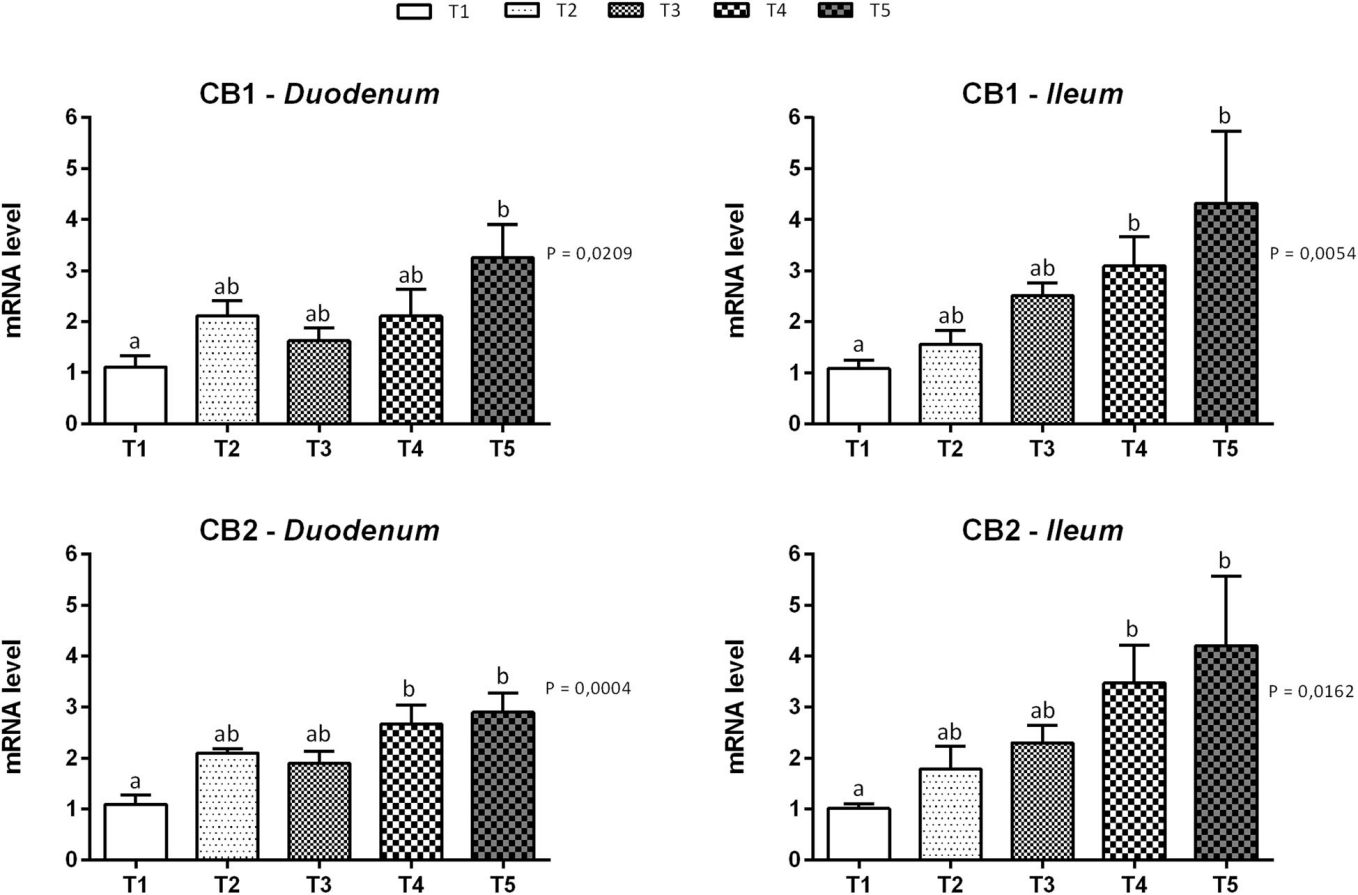
Gene expression of cannabinoid receptors in duodenal and ileal mucosa of piglets. Data are expressed as means (*n* = 8) and S.E.M. represented by vertical bars. ^a, b^ Values with different superscripts differ significantly at *P* < 0.05. T1 = basal diet; T2 = basal diet + 25.5 mg of thymol/kg feed; T3 = basal diet + 51 mg of thymol/kg feed; T4 = basal diet + 153 mg of thymol/kg feed; T5 = basal diet + 510 mg of thymol/kg feed (Vetagro SpA, Reggio Emilia, Italy). A modification of the 2^–ΔΔCT^ method was used to analyze the relative expression (fold changes), calculated relative to the control group (control; Livak and Schmittgen, 2001 [21]). CB1 = cannabinoid receptor 1; CB2 = cannabinoid receptor 2

**Fig. 2:**
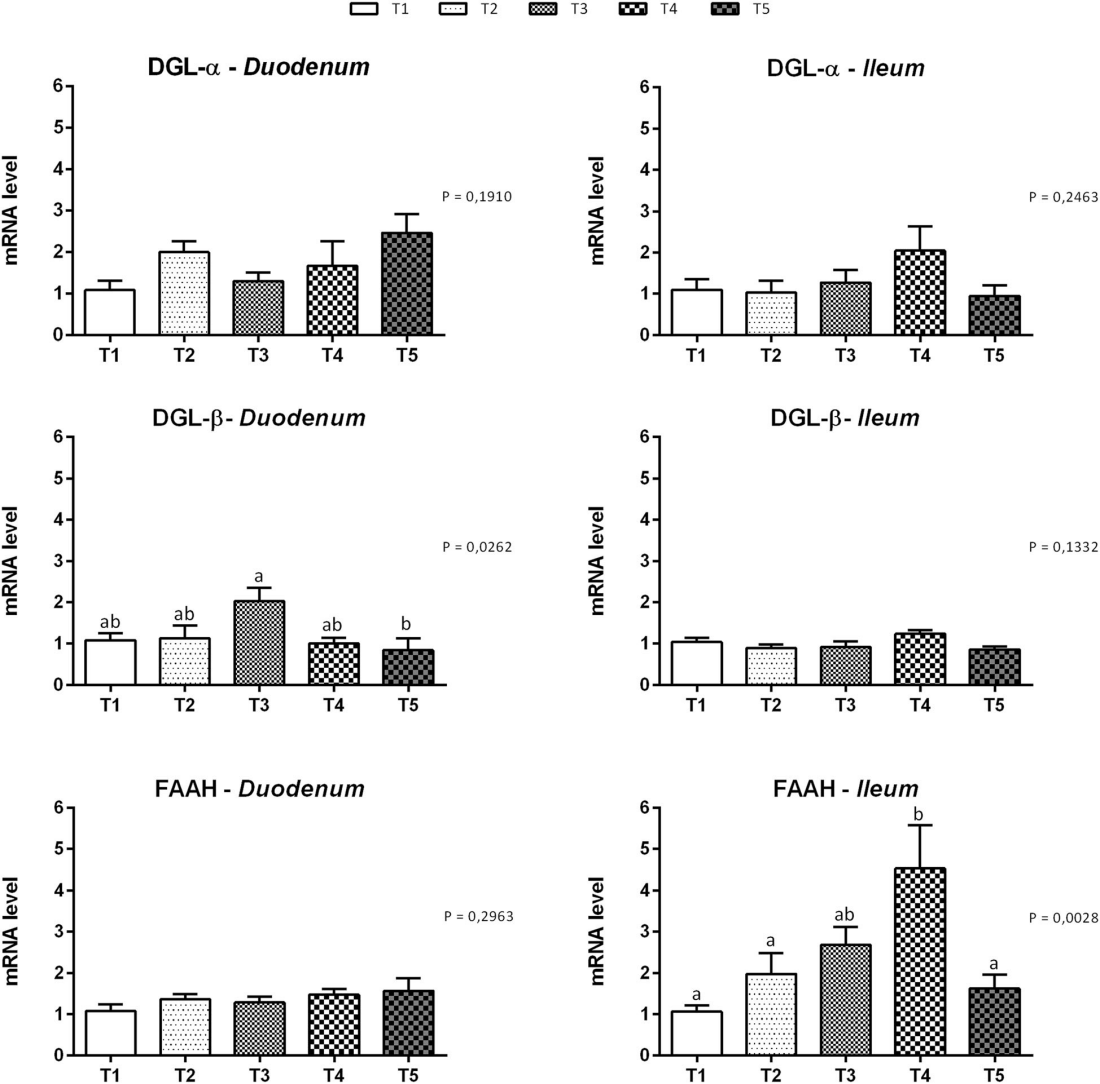
Gene expression of endocannabinoid enzymes in duodenal and ileal mucosa of piglets. Data are expressed as means (*n* = 8) and S.E.M. represented by vertical bars. a, b Values with different superscripts differ significantly at *P* < 0.05. T1 = basal diet; T2 = basal diet + 25.5 mg of thymol/kg feed; T3 = basal diet + 51 mg of thymol/kg feed; T4 = basal diet + 153 mg of thymol/kg feed; T5 = basal diet + 510 mg of thymol/kg feed (Vetagro SpA, Reggio Emilia, Italy). A modification of the 2–ΔΔCT method was used to analyze the relative expression (fold changes), calculated relative to the control group (control; Livak and Schmittgen, 2001 [21]). DGL-α = diacylglycerol lipase alpha; DGL-β = diacylglycerol lipase beta; FAAH = fatty acid amide hydrolase

Figure 1 summarizes gene expression data for cannabinoid receptors in the duodenal and ileal mucosa at d14. Cannabinoid receptor 1 and 2 mRNAs were detected in both the duodenal and ileal mucosa. The level of the CB1 mRNA was significantly increased in the duodenum of the T5 group (*P* = 0.0209) and in the ileum of the T4 and T5 groups (*P* = 0.0054) compared to the control group. Significantly increased levels of the CB2 mRNA were detected in both the duodenum and ileum of groups T4 and T5 compared to the control group (*P* = 0.0004 and *P* = 0.0162 respectively). Data on gene expression for ECS enzymes are reported in Fig. 2. The presence of mRNA for all the enzymes tested was confirmed. The expression of the DGL-α mRNA in both duodenum and ileum was not affected by the treatments. The expression of the DGL-β mRNA was significantly increased in the duodenum of animals fed 51 mg of thymol/kg of feed (T3) compared to animals fed 510 mg of thymol/kg of feed (*P* = 0.0262). No significant differences were identified in the levels of the DGL-β mRNA in the ileal mucosa. Differences in the levels of the FAAH mRNA were not observed in the duodenum, while mRNA levels were significantly increased in the ileum of the T4 group compared to the control group (*P* = 0.0028).

### Gut chemosensing system

**Fig. 3:**
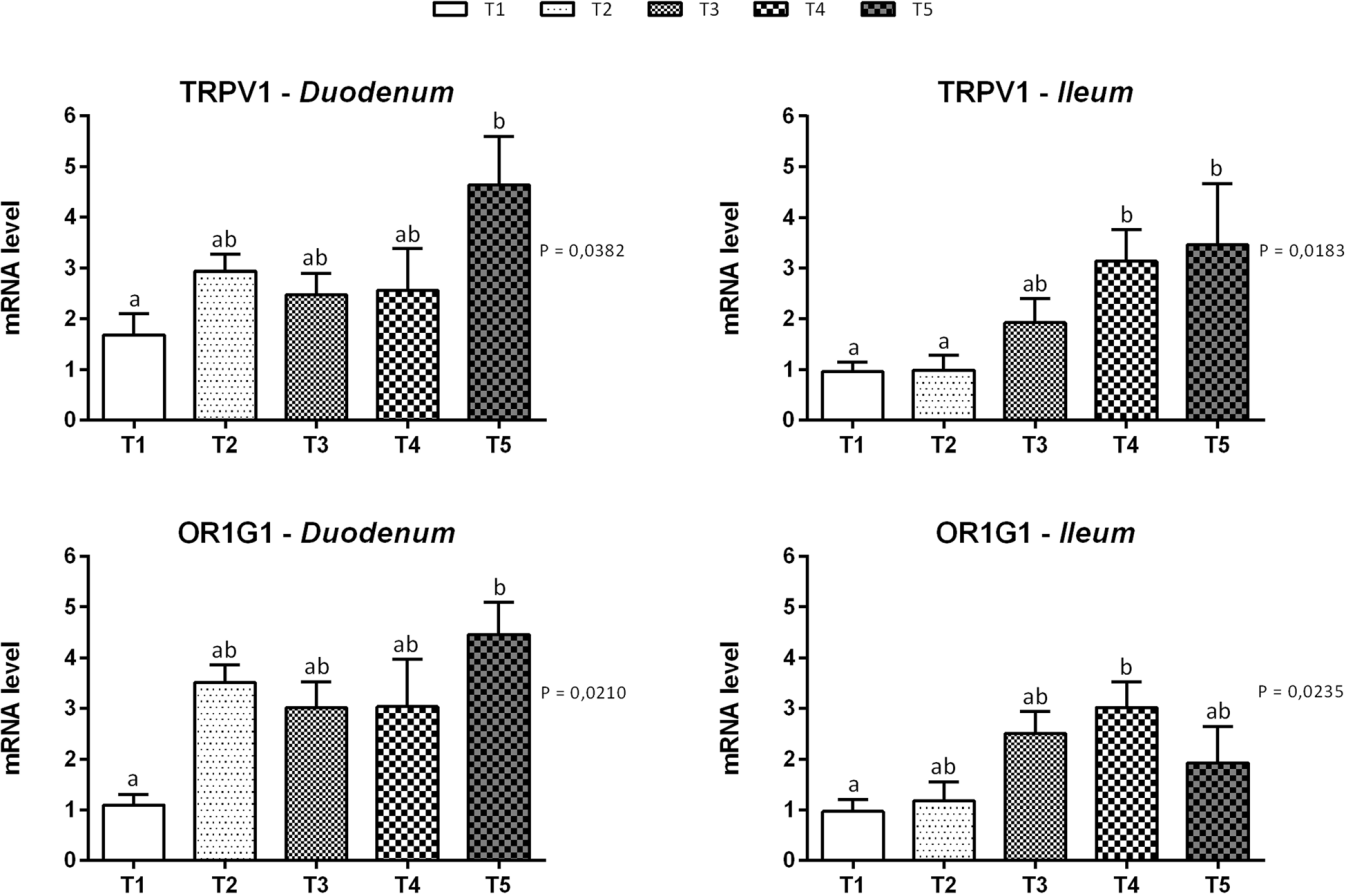
Gene expression of chemosensory receptors in duodenal and ileal mucosa of piglets. Data are expressed as means (*n* = 8) and S.E.M. represented by vertical bars. a,b Values with different superscripts differ significantly at *P* < 0.05. T1 = basal diet; T2 = basal diet + 25.5 mg of thymol/kg feed; T3 = basal diet + 51 mg of thymol/kg feed; T4 = basal diet + 153 mg of thymol/kg feed; T5 = basal diet + 510 mg of thymol/kg feed (Vetagro SpA, Reggio Emilia, Italy). A modification of the 2–ΔΔCT method was used to analyze the relative expression (fold changes), calculated relative to the control group (control; Livak and Schmittgen, 2001 [21]). TRPV1 = transient receptor potential vanilloid 1; OR1G1 = Olfactory receptor 1G1

Results for the gut chemosensing are reported in Fig. 3. Concerning the gut chemosensing markers, both the TRPV1 and OR1G1 (Olfactory receptor 1G1) mRNAs were detected in the duodenal and ileal mucosa. Moreover, the supplementation of 510 mg of thymol/kg of feed increased the level of the TRPV1 mRNA in the duodenum (*P* = 0.0382), while increased mRNA levels were observed in the ileum of the T4 and T5 groups compared to the control group (*P* = 0.0183). The OR1G1 mRNA was expressed at higher levels in the duodenum of animals provided feed supplemented with 510 mg of thymol/kg of feed (T5) (*P* = 0.0210) and in the ileum of animals fed 153 mg of thymol/kg of feed (T4) (*P* = 0.0235) than in the control group.

### Inflammatory cytokines

**Fig. 4:**
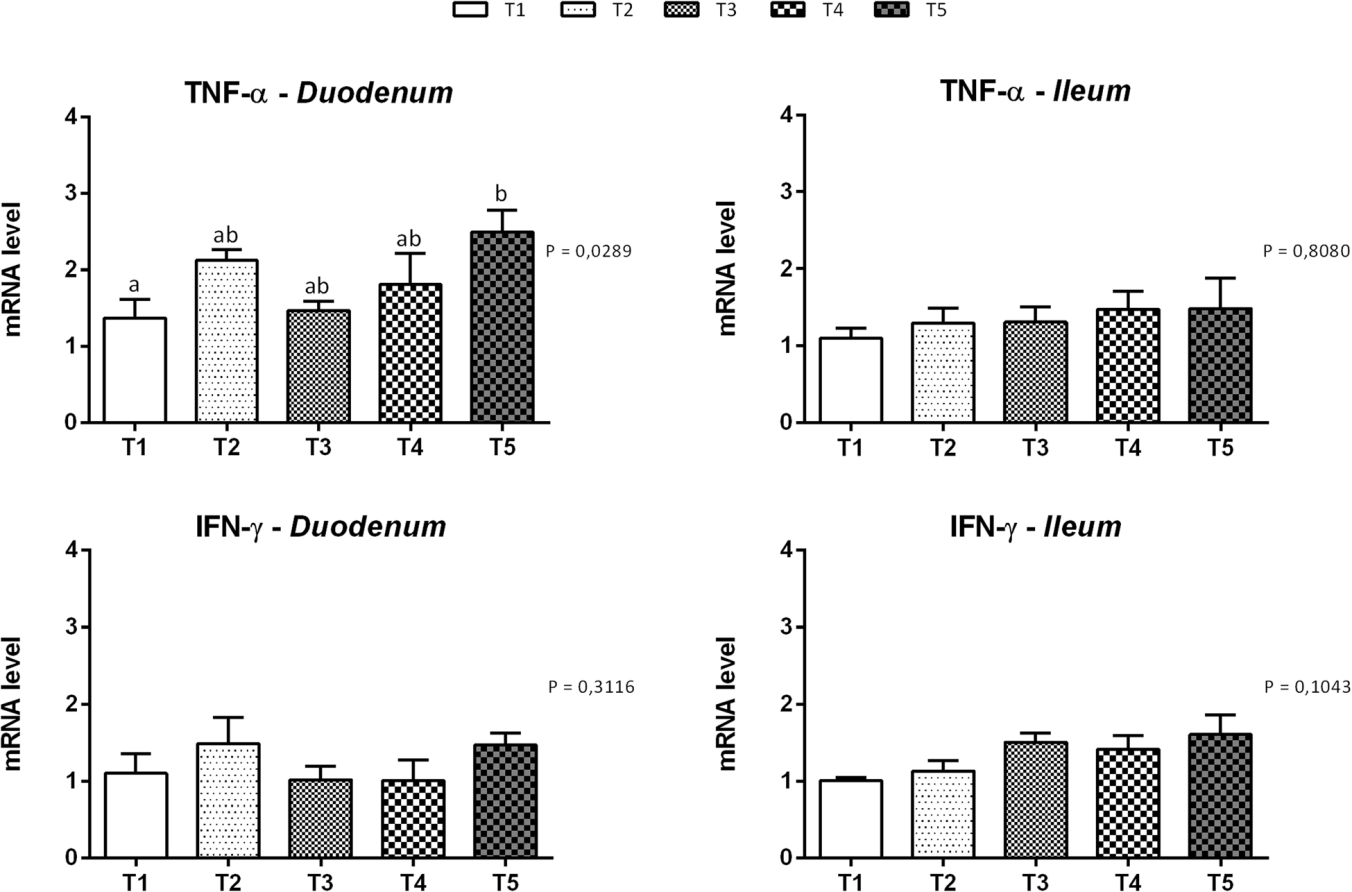
Gene expression of inflammatory cytokines in duodenal and ileal mucosa of piglets. Data are expressed as means (*n* = 8) and S.E.M. represented by vertical bars. a, b Values with different superscripts differ significantly at *P* < 0.05. T1 = basal diet; T2 = basal diet + 25.5 mg of thymol/kg feed; T3 = basal diet + 51 mg of thymol/kg feed; T4 = basal diet + 153 mg of thymol/kg feed; T5 = basal diet + 510 mg of thymol/kg feed (Vetagro SpA, Reggio Emilia, Italy). A modification of the 2–ΔΔCT method was used to analyze the relative expression (fold changes), calculated relative to the control group (control; Livak and Schmittgen, 2001 [21]). TNF-α = tumor necrosis factor-α; IFN-γ = interferon-γ

**Fig. 5:**
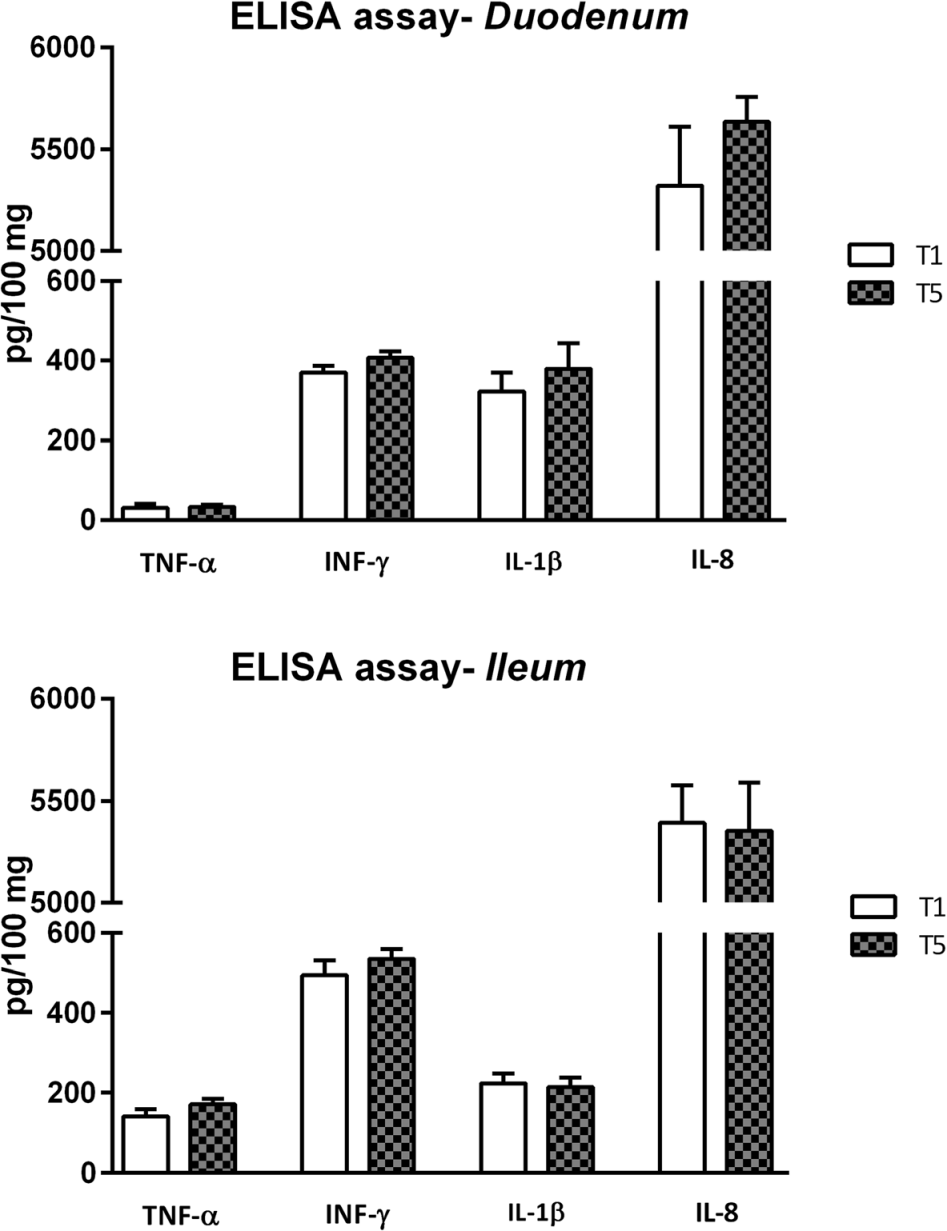
Protein expression of inflammatory cytokines in duodenal and ileal mucosa of piglets. Data are expressed as means (*n* = 8) and S.E.M. represented by vertical bars. Data refer to picograms of cytokine per 100 mg of tissue (pg/100 mg). T1 = basal diet; T5 = basal diet supplemented with 510 mg of thymol / kg feed (Vetagro SpA, Reggio Emilia, Italy). TNF-α = tumor necrosis factor-α; IFN-γ = interferon-γ; IL-1β = interleukin-1β; IL-8 = interleukin-8

Figures 4 and 5 show the mRNA and protein levels of inflammatory cytokines in the duodenal and ileal mucosa at d14, respectively. Significantly increased levels of the Tumor necrosis factor (TNF)-α mRNA were detected in the duodenum of animals fed thymol; in particular, the T5 group displayed the highest expression of TNF-α (*P* = 0.0289). On the other hand, the level of the TNF-α mRNA in the ileal mucosa was not affected by the treatments. No difference was reported in interferon (IFN)-γ expression in both the duodenum and ileum. No statistically significant differences in the levels of these proteins were observed among the treatment groups (Fig. 5).

## Discussion

Over the last 50 years, the ability of the ECS to modulate the inflammatory status has been investigated in human medicine, due to the pharmacological potential of cannabinoid therapy to treat clinical conditions such as IBD, Crohn’s disease and ulcerative colitis [22]. Similarly, the gut chemosensory system also shows potential as a target for IBD, gluten sensitivity, and obesity [23]. In this paper, we focused our attention on the presence of ECS and gut chemosensing markers in the duodenal and ileal mucosa of piglets, with a particular focus on both the receptors, namely, CB1, CB2, TRPV1 and OR1G1, and the main enzymes involved in the synthesis and degradation of AEA and 2-AG by performing a molecular analysis. To our knowledge, this paper is the first to describe the alleged roles of the ECS and gut chemosensing system on the intestinal functionality of swine and their possible modulation by thymol.

Interesting results were obtained from the analysis of gene expression of ECS markers, as we detected mRNA for all of these markers. Since it was first described in the central nervous system, the ECS is now proposed to regulate different physiological mechanisms. More information is now available about this signaling system, but little evidence is available for its role in the organism. Recently, an increasing number of papers examining the role of the ECS in various pathologies has been published, but the possible roles of ECS in animal medicine, production and nutrition have not been investigated in depth. Notably, the activation of CB1 receptors is involved in inflammation and cell death in different experimental models of disease [22], but evidence for a possible role of nutrition in modulating this system has not been available until now. Interestingly, we detected the CB1 and CB2 mRNAs in mucosa scrapings, confirming the presence of these receptors in the mucosa of piglets and suggesting that these receptors are not exclusively localized in the ENS. The classically recognized location of the endocannabinoid receptors is in the ENS, and the only report analyzing their presence on the mucosa was performed in mice. In mouse, Sykaras and colleagues [24] described the presence of the CB1 mRNA in the EEC, where it is suggested to drive the intake of fat-rich foods by inhibiting the release of cholecystokinin (CCK), which normally induces satiation after meals. In addition, CB1 activation inhibits acetylcholine (Ach) and is accompanied by a reduction in GI muscle contraction, resulting in decreased intragastric pressure and the inhibition of gastric emptying, pyloric contraction, and intestinal motility [11].

Cannabinoid receptor 1 also regulates food intake in rats, particularly during starvation periods, by increasing 2-AG synthesis through the activity of DGL [25, 26], suggesting a possible role during weaning. Thymol appears to modulate the expression of these receptors and enzymes. The trend in the use of botanicals in animal feed has increased during the last two decades. Thymol is one of the most well-studied and frequently used molecules in animal nutrition due to its countless beneficial properties [27]. Considering the increase in the levels of the CB1 and CB2 mRNAs in both the duodenum and ileum, we postulated that thymol stimulates the synthesis of the receptors in the GI tract. Cannabinoid receptor 1 and cannabinoid receptor 2 are recognized to play a protective role in IBD, namely, by functioning to control the inflammatory status and are normally present during weaning and starvation [28]. Therefore, the ECS may play a role in the response to stressful situations, such as weaning for piglets, and thymol potentially exerts a positive effect by stimulating the expression of endocannabinoid receptors. Unfortunately, no studies examining this topic have been published to date, but the use of thymol may plausibly support piglets during weaning by activating the ECS.

Additionally, levels of the DGL and FAAH mRNAs were increased; as mentioned above, DGL is involved in the synthesis of 2-AG, while FAAH is responsible for AEA degradation. According to Bashashati and colleagues [29], the inhibition of the degradation of 2-AG decreased whole-gut transit in mice; conversely, Izzo and colleagues [30] concluded that a decrease in the small intestinal content of AEA leads to an increase in transit, suggesting that 2-AG modulates CB1-controlled gut contractility. Thymol may also play a role in modulating gut contractility by regulating enzyme expression and, consequently, the concentrations of the bioactive lipids, resulting in the modulation of gut contractility.

Other interesting data include the detection of mRNAs encoding receptors involved in the gut chemosensory system. Again, we not only detected the TRPV1 and OR1G1 mRNAs in piglet mucosa scrapings but also observed increased levels of the TRPV1 and OR1G1 mRNAs when the diet was supplemented with thymol. The colonic mucosa of both humans and rats express OR mRNAs, and luminal odorants induce serotonin secretion in isolated duodenal enterochromaffin cells and enterochromaffin cell lines [31]. Moreover, the activation of OR1G1 by luminal thymol increases [Ca^2+^]_i_. The elevated [Ca^2+^]_i_ may modulate Ca^2+^-activated basolateral K^+^ channels, providing a driving force for the exit of Cl^−^/HCO_3_^−^ [32]. As result, thymol activates certain types of the apical odorant receptor OR1G1 and stimulates Cl^−^ secretion in colonic epithelial cells. Recently, human OR1G1 was reported to participate in glucose homeostasis during meal ingestion by inducing gut peptide secretion [33]. A reasonable conclusion is that OR1G1 may play a role in controlling intestinal permeability and nutrient absorption, although only a few studies examining the role of this OR are available, and, moreover, its presence in the swine GI tract is not well documented. Considering that in this study the mRNA of OR1G1 was detected in both duodenum and ileum, it is clear that this receptor, and probably other receptors of this class, are also present in the pig GI tract.

Thymol has also been linked to the immune system due to its anti-inflammatory and antioxidant properties [19]. Omonijo et al. [34] reported a reduction in LPS-induced ROS (reactive oxygen species) and TNF-α levels in cells pretreated with thymol. In the present study, piglets fed microencapsulated thymol exhibited increased levels of the TNF-α mRNA; this result might appear surprising because this cytokine has a pro-inflammatory function, but no adverse effects on the piglets’ health were observed. Moreover, the level of the TNF-α protein was not altered by the treatments. Interestingly, at the dose where we observed an increase in the level of the TNF-α mRNA, we also registered an increase in the expression of the CB1 and CB2 mRNAs. The cannabinoid receptors act on TNF-α in two opposite directions. Stimulation of CB1 reduces TNF-α release from activated microglia [35]; conversely, CB2 is known to induce the expression of pro-inflammatory cytokines to promote a TH_1_ immune response [36], which may explain the increase in the gene expression of this cytokine. Finally, thymol increases the levels of the CB1 and CB2 mRNAs, which controls the release and the expression of TNF-α, respectively, providing an explanation for the regulation of the protein and mRNA levels of this pro-inflammatory cytokine. Moreover, the absence of variations in terms of protein levels of inflammatory cytokines obtained with the ELISA assay is not unexpected, considering that all the piglets were healthy and, without a specific challenge or stressors, pro-inflammatory and anti-inflammatory cytokines are normally in equilibrium [37].

Concerning the zootechnical performance, in this study the administration of thymol to weaned piglets at different levels of inclusion did not modify any parameters. Thymol was well tolerated by the animals also at the higher level of inclusion (10x the highest recommended dose in T5 group). This is an interesting result, mainly regarding FI and ADG, which are normally reduced when thymol is supplemented at a high dosage [38–40]. This high tolerability of thymol could be explained thanks to the microencapsulation of the terpene in a lipid matrix. It is well documented that encapsulation technologies are useful not only to deliver the bioactive compound along all the GI tract [41–43], but also to increase the palatability of some pungent molecules, such as thymol [44].

Despite the increasing number of studies analyzing the roles of ECS and chemosensation in the organism, additional studies are required to understand how endocannabinoid receptors and molecules control various pathologies and inflammation. Nevertheless, these results represent a first insight into the potential uses of botanical feed additives to control intestinal inflammation and motility. Further investigations are required to obtain a better understanding of the protein expression and localization of these markers, together with the possible mechanism by which thymol modulates the expression of endocannabinoid markers.

## Conclusions

In conclusion, our data not only confirm the presence of the ECS and gut chemosensing markers in the duodenal and ileal mucosa of piglets but also suggest that thymol modulates the gene expression of these markers. Thymol increases the expression of the mRNAs encoding the CB1 and CB2 receptors both in the duodenum and ileum. This compound also modulates the mRNA levels of enzymes involved in the biosynthesis and degradation of endocannabinoid molecules. Moreover, the upregulation of OR1G1 and TRPV1 by thymol throughout the intestine implicates a possible role for these receptors as mediators of the effects of thymol as a feed additive on promoting gut health.

## Methods

### Ethics statement

The study was conducted at the facilities of the Research Center for Animal Production and Environment (CERZOO), which is Good Laboratory Practices-certified and operates according to the Procedure of Animal Protection and Welfare (Directive No 86/609/EEC). Animals used in the study were raised and treated according to European Union Directive 2010/63/EU. The study was authorized by Italian Health Ministry according to art. Thirty-one of the Italian Legislative Decree No. 26/2014 and to the recommendation of Commission 2007/526/CE, covering the accommodation and care of animals used for experimental and other scientific purposes (authorization n. 341/2017-PR issued on May 3, 2017). Animals were obtained from a breeding farm in Cascina Mandellina, Bergamo, Italy.

### Animals and diets

One hundred sixty piglets (Duroc × Large White) weaned at 28 days of age and with a BW of 7.71 ± 1.00 kg were divided into 40 pens (4 piglets per pen, castrated males and females were blocked in separate pens) and randomly assigned to one of the following experimental groups (*n* = 32): control group fed the basal diet (T1), a group fed the basal diet supplemented with 25.5 mg of thymol/kg feed (T2), a group fed the basal diet supplemented with 51 mg of thymol/kg feed (T3), a group fed the basal diet supplemented with 153 mg of thymol/kg feed (T4) and a group fed the basal diet supplemented with 510 mg of thymol/kg feed (T5). Thymol was provided in a form microencapsulated in a lipid matrix (Vetagro SpA, Reggio Emilia, Italy). Concentrations of thymol were selected to meet or exceed the upper limit of inclusion in food and feed established by the European Agency for the Evaluation of Medicinal Products [45, 46]. The basal feed was formulated to meet or exceed the nutritional requirements of pigs according to National Research Council [47], and feed and water were provided ad libitum (the composition of the basal diet is reported in Table 1). The health status of animals was monitored throughout the study. Piglets were individually weighed at the beginning (day 0) and end of the study (day 14). Growth parameters, such as FI, ADFI, ADG, and F:G, were measured in the animals housed in each pen on d14 of the experiment.

**Table 1:** Basal diet composition (%) and nutrient profile

Ingredient (% of dry matter)	
Corn meal	59.25
Soybean meal, 44%	21.90
Sweet milk whey	8.00
Fish meal (Herring 999)	7.00
Soybean oil	1.98
Calcium carbonate	0.35
Vitamin and mineral premix^1^	0.25
L-Lysine HCl	0.54
NaCl	0.16
L-Threonine	0.24
DL-Methionine	0.26
L-Tryptophan	0.08
Total	100
Calculated nutrient composition	
Digestible Energy^2^ (kcal/kg feed)	3523
Net Energy^2–3^ (kcal/kg feed)	2549
Dry matter (%)	88.53
Crude protein (%)	21.00
Crude fiber (%)	2.64
Crude fat (%)	5.32
Calcium (g/kg)	7.00
Phosphorus – Total (g/kg)	5.60
Phosphorus – Available (g/kg)	1.45
Sodium (g/kg)	1.80

^1^Content of vitamins and Oligo minerals/kg feed: vit. A: 10,000. UI; vit. D3: 1000UI; vit. E: 100 mg; vit. B1: 3.0 mg; vit. B2: 10.0 mg; vit. B6:5.8 mg; vit. B12: 0.04 mg; Biotin: 0.19 mg; vit. K: 4.8 mg; vit. PP: 35.0 mg; folic acid: 1.4 mg; D-pantothenic acid: 26.1 mg; choline chloride: 120 mg; 49.7 mg Mn from Manganese oxide; 224 mg Fe from Ferrous carbonate; 75.8 mg Cu from Copper sulphate pentahydrate; 139 mg Zn from Zinc oxide; 0.89 mg I from Calcium iodide; 0.64 mg Se from Disodium selenite

^2^Aritmetic mean

^3^Net Energy was calculated according to the procedure and equation proposed by Noblet and colleagues [48]

At the end of the study, 8 animals from each treatment group were selected for sacrifice, sample collection, and analysis. Piglets were euthanized by a penetrating captive bolt followed by exsanguination. Duodenal and ileal mucosal scrapings were collected. The duodenum and ileum were longitudinally cut to expose the mucosa, washed with a phosphate-buffered saline solution to remove mucus and digesta, then scraped gently with a glass slide, packed, immediately frozen in liquid N_2_ and stored at − 80 °C until the analyses of gene and protein expression.

### Gene expression analysis

Gene expression was analyzed using the method reported by Herfel et al. [49]. Duodenal and ileal scraping samples obtained on d14 of the study were disrupted by grinding them in liquid nitrogen with mortar and pestle, and then homogenized using a TissueLyser (Qiagen, Hilden, Germany). Total RNA was isolated using a NucleoSpin® RNA Kit (Macherey-Nagel, Düren, Germany) according to the manufacturer’s instructions. Genomic DNA contamination was removed by treating the samples with the deoxyribonuclease supplied in the extraction kit (rDNase, RNase-free; Macherey-Nagel). The RNA yield and quality were determined spectrophotometrically by measuring the absorbance at 260 and 280 nm (A260 and A280 nm, respectively) (Microvolume Mode with SmartPath® Technology, Denovix). One microgram of RNA was reverse transcribed with the iScript cDNA Synthesis Kit (Bio-Rad Laboratories Inc., Hercules, CA, USA) according to the manufacturer’s instructions. Real-time PCR was performed using an iCycler Thermal Cycler system and SybrGreen Supermix (Bio-Rad Laboratories Inc.). The thermocycling protocol included an initial denaturation step for 1 min and 30 s at 95 °C, followed by 40 cycles of denaturation at 95 °C for 15 s and 30 s of annealing and extension at 60 °C. After amplification, a melting curve analysis was performed for all the samples, with slow heating from 55 °C to 95 °C at a rate of 0.5 °C/s to validate the absence of non-specific products. Gene expression was normalized to a housekeeping gene (HK) encoding portions of porcine ribosomal subunit 60 S, in particular ribosomal protein L35 (RPL35). The average threshold cycle (CT) was determined for each gene of interest, and the geometric average was calculated for HK by assuming that CT is the number of cycles needed to reach a fixed arbitrary threshold. Delta CT was calculated, then a modification of the 2^–ΔΔCT^ method [21] was used to analyze the relative expression (fold changes), which was calculated relative to the control group. The sequences, accession numbers in the EMBL database/GenBank, expected product lengths and references for porcine primers are provided in Table 2. Primer oligonucleotides for CB1, DGL-β and OR1G1 were designed using the Primer-BLAST tool (NCBI National Center for Biotechnology Information, www.ncbi.nlm.nih.gov). Primers were obtained from Life Technologies (Life Technologies Italia).

**Table 2:** Primer sequence used for gene expression analysis

Gene	Primer sequence (F and R) 5′ → 3′	Product length (bp)	Accession N.	Reference
CB1	F: TTCCCCACTTCTTTTCCGCC R: GGGAGTCCCTTCGCATCC	208	XM_013992672.2	Present study
CB2	F: TTTATAGCCTGGCCTCCCCT R: TTTTCCCGTCTGCCTCTGTC	240	XM_021095530.1	[50]
FAAH	F: TGCCACCGTGCAAGAAAATG R: CCACTGCCCTAACAACGACT	234	XM_013999418.2	[50]
DGL-α	F: GAAACCAAACACGCCTCCAC R: CAACCCAGCAGCAAAGGAAC	211	XM_021082924.1	[50]
DGL-β	F: TTTGTAATCCCGGACCACGG R: GACCTGCCGAGGAATACGGA	255	XM_021086077.1	Present study
TRPV1	F: TCACCAACAAGAAGGGGCTC R: GGATAGGTGCCTGCACTCAG	116	XM_005669121.1	[51]
OR1G1	F: CTTGGTTTGTGTGCTCTGCC R: GAAAAGGCTTTCCGCTTCCC	96	XM_013990010.1	Present study
INF-γ	F: GGCCATTCAAAGGAGCATGGATGT R: TGAGTTCACTGATGGCTTTGCGCT	149	NM_213948.1	[52]
TNF-α	F: GCCCACGTTGTAGCCAATGTCAAA R: GTTGTCTTTCAGCTTCACGCCGTT	99	NM_214022.1	[52]
RPL35	F: AACCAGACCCAGAAAGAGAAC R: TTCCGCTGCTGCTTCTTG	146	NM_214326.2	[53]

*F* forward, *R* reverse, *CB1* cannabinoid receptor 1, *CB2* cannabinoid receptor 2, *FAAH* fatty acid amide hydrolase, *DGL-α* diacylglycerol lipase alpha, *DGL-β* diacylglycerol lipase beta, *TRPV1* transient receptor potential vanilloid 1, *OR1G1* olfactory receptor 1G1, *IFN-γ* interferon-γ, *TNF-α* tumor necrosis factor-α, *RPL35* ribosomal protein L35.

### ELISA quantification of inflammatory cytokine concentrations

Duodenal and ileal mucosal scrapings were disrupted by grinding the samples in liquid nitrogen with a mortar and pestle, followed by the addition of lysis buffer (10 mM-2-amino-2-hydroxymethyl-propane-1,3-diol (Tris)-HCl, 1 mM-ethylenediaminetetraacetic acid (EDTA), and 0.5%-Triton X100) and homogenization using a TissueLyser (Qiagen). Protein levels of inflammatory cytokines (TNF-α, INF-γ, IL-1β, and IL-8) were analyzed using ELISA kits specific for porcine cytokines (Quantikine ELISA, R&D Systems Inc., Minneapolis, MN, USA). Analyses were performed according to the manufacturer’s instructions. ELISA quantification was performed for samples obtained from the T1 and T5 groups. Results are reported as picograms of cytokine per 100 mg of tissue (pg/100 mg).

### Statistical analysis

Animals were blocked in a completely randomized design and data were analyzed using GraphPad Prism® software (GraphPad Software, Inc., La Jolla, CA, USA). Data were analyzed using one-way ANOVA followed by Tukey’s post hoc test to detect differences among treatments. The pen was the experimental unit for growth performance, whereas the pig was the experimental unit for gene expression and ELISA data. Differences were considered significant at *P* < 0.05, and trends were defined at 0.05 ⩽ *P* < 0.1.

## Data Availability

The datasets used and/or analyzed during the current study are available from the corresponding author on reasonable request. The sequences used for the primer design are available in the Genbank repository (https://www.ncbi.nlm.nih.gov/genbank/). All accession numbers are listed in Table 2.

## Abbreviations

ECS: Endocannabinoid System
GPCRs: G protein-coupled receptors (GPCRs)
CB1: Cannabinoid receptor 1
CB2: Cannabinoid receptor 2
TRPV1: Transient receptor potential vanilloid 1
AEA: Anandamide
2-AG: 2-arachidonoylglycerol
NAPE-PLD: N-Acylphosphatidylethanolamine-specific Phospholipase D
DGL: Diacylglicerol Lipase
FAAH: Fatty Acid Amide Hydrolase
MAGL: Monoacylglicerol Lipase
GI: Gastrointestinal
ENS: Enteric Nervous System
LPS: Lipopolysaccharide
IBD: Irritable Bowel Disease
EEC: Enteroendocrine Cells
TRP: Transient Receptor Potential
ORs: Olfactory Receptors
BW: Body Weight
FI: Feed Intake
ADFI: Average Daily Feed Intake
ADG: Average Daily Gain
F: G: Feed to Gain ratio
OR1G1: Olfactory receptor 1G1
TNF-α: Tumor Necrosis Factor-α
IFN-γ: Interferon-γ
CCK: Cholecystokinin
Ach: Acetylcholine
ROS: Reactive Oxygen Species
HK: Housekeeping
CT: Threshold cycle
RPL35: Ribosomal protein L35
EDTA: Ethylenediaminetetraacetic acid
